# Biofilms and their role on diseases

**DOI:** 10.1186/s12866-023-02954-2

**Published:** 2023-07-31

**Authors:** Vijay Singh Gondil, Bindu Subhadra

**Affiliations:** 1 Department of Microbiology and Immunology, University of Rochester Medical Center, Rochester, NY 14642 USA; 2 College of Veterinary Medicine, Long Island University, Brookville, NY 11548 USA

## Abstract

Biofilms are complex, three-dimensional structures that provide a long-established survival mechanism for microorganisms. Biofilms play a substantial role in pathogenesis as they can evade the immune response and be highly resistant to conventional antimicrobials, thus impacting the human health and healthcare system. To address this issue, *BMC Microbiology* invites submissions to the collection ‘Biofilms and its impact on disease’.

Biofilms are formed by communities of microorganisms (bacteria, fungi, or protists) that are embedded in self-produced, extracellular polymeric substances (EPS) [1]. The EPS comprise polysaccharides, extracellular DNAs, and secreted proteins [1]. The formation of biofilms protects the microorganisms against the host immune system, often helping them to grow and establish chronic infections [2]. In addition, the pathogenic microbes in biofilms are highly resistant to antimicrobials, making their infections difficult to treat [3]. As biofilm-associated infections become more and more prevalent, it is of utmost importance to understand various aspects of biofilm formation and the functionality of biofilms, which will aid to develop strategies to tackle these infections.

Bacterial biofilm formation is a well-regulated, multi-step process involving (i) attachment, (ii) EPS production, (iii) biofilm maturation, and (iv) biofilm dispersal/detachment [4]. Both inert and biological surfaces can be substrates for the initial bacterial attachment, which can be reversible or irreversible. Once the bacteria are attached to the surfaces irreversibly, EPS is synthesized by the attached cells using the cell-to-cell communication mechanism known as quorum sensing (QS) [5]. EPS play a vital role in biofilm structure, signaling, trapping of nutrients and water, and genetic exchange, among other processes [5]. Apart from proteins such as enzymes and proteinaceous structures like pili and fimbriae, EPS also contain lipids which are essential during the attachment step [5]. During biofilm maturation, microcolonies are formed, water-filled circulatory systems are produced, and gene expression is altered extensively through the QS signaling molecules [5]. Biofilm dispersal is a strategy of bacteria to leave biofilms and continue their life in a new substratum. Biofilm dispersal has been shown to play a crucial role in spreading the disease within the host, and in horizontal and vertical cross-host transmission [6].

Though biofilms can have positive functions, as reported for the commensal organism, *Staphylococcus epidermidis*, while preventing colonization of pathogenic bacteria [7], most biofilms are associated with infections and diseases. In healthcare settings, biofilm-forming bacteria can grow on medical devices (e.g. catheters, prosthetic heart valves, pacemakers, breast implants, contact lenses and cerebrospinal fluid shunts), as well as on dead and/or living tissues. The bacteria most frequently reported in biofilms populating such devices are *S. aureus*, *S. epidermidis* and *Pseudomonas aeruginosa* [7]. *P. aeruginosa* can also form biofilms in the water distribution systems of health care settings [7]. Among various biofilm-associated infections and diseases, notorious examples include cystic fibrosis (*P. aeruginosa*), otitis media (*Haemophilus influenzae*), periodontitis (*P. aerobicus* and *Fusobacterium nucleatum*), infective endocarditis (*S. aureus*, *Viridans streptococci*, and *Enterococcus faecalis*), chronic wounds (*P. aeruginosa*) and osteomyelitis (*P. aeruginosa*) [7]. It is reported that the majority (65%) of the infectious agents are associated with biofilm production, and they display high resistance to antimicrobials (up to 1000 folds) and components of the host immune system, making them extremely difficult to treat.

Bacterial biofilms are responsible for the majority of chronic antibiotic resistance infections, which are difficult to cure with conventional antibacterial agents [2]. Due to the rapid emergence of antibiotic resistance and the slow pace of development of newer antibiotics, a variety of natural and synthetic alternative antibacterial agents are being explored. Various natural products such as lantibiotics (nisin, subtilin, epidermin), antimicrobial peptides (LL-37, Burford-II, PR-39), phytochemicals (tannins, flavonoids, flavones, flavonols), bacteriophages and enzymes (DNases, depolymerases, lactonases, and bacteriophage-based endolysins) have been extensively studied for the inhibition of biofilm formation [8]. Moreover, synthetic molecules such as sodium citrate, ethylenediaminetetraacetic acid, metallic nanoparticles (silver, zinc, copper), cadexomer iodine and chlorhexidine have also been exploited as potent anti-biofilm agents [9]. Various mechanisms are involved in the anti-biofilm activity of these alternative agents, which consists of the inhibition of the QS pathway, disruption of extracellular matrix, inhibition of stringent bacterial response, biofilm disassembly, increased membrane permeabilization, inhibition of signaling pathway and neutralization of lipopolysaccharides [10]. Moreover, there are reports on anti-biofilm molecules (such as esculetin and octenidine hydrochloride) showing their effective anti-biofilm activity, despite their mechanism of action is still unknown and needs to be further explored before achieving vast therapeutic applications [9]. These anti-biofilm agents may exert their antibacterial activity in combination with anti-biofilm molecules and in the presence of conventional antibiotics, leading to increased susceptibility to the available treatment options. It is hypothesized that combining more than one anti-biofilm agent from different sources might interfere with varying stages of biofilm development to exert a higher anti-biofilm activity, with lower chances of developing selective pressure-mediated resistance [11]. Furthermore, the usage of anti-biofilm agents on implant surfaces is also employed as a preventive strategy to counter implant-associated bacterial infections. In recent studies, various molecules such as nisin, endolysins, chitosan, algal polysaccharide ulvan, dextran and dermatan sulfate showed their anti-biofilm efficacy on biomedical surfaces [8, 12].

Biofilm-associated infections and the emergence of antibiotic resistance have elevated the need for novel therapeutic agents and their expedited regulatory approvals. Despite discovering and exploring numerous anti-biofilm agents, these molecules are still limited to *in-vitro* and *in-vivo* animal infection studies because of the poor knowledge of their pharmacokinetic and pharmacodynamic properties. In this context, understanding the mechanism of action of anti-biofilm agents provides a roadmap to improve the efficacy of the drug by incorporating chemical modifications or using combinational therapy. Until now, various anti-biofilm agents have shown their potency in preclinical studies [8], while there is a need for an increased number of phase 1–4 clinical trials to validate the safety and efficacy of these compounds in human subjects. Currently, ongoing clinical trials for anti-biofilm agents are primarily focused on oral biofilms; however, evaluating the safety and effectiveness of these agents in systemic and deep-located biofilm infections is also very relevant.

As our understanding of the mechanism of action and clinical effectiveness of anti-biofilm agents continues to expand, we anticipate that new and effective alternatives will be developed to improve the clinical treatment of biofilm-associated infections. Here we summarize some of the past and ongoing research challenges on biofilms, from a disease and infection perspective. We invite researchers to submit their studies to the collection ‘Biofilms and its impact on disease’, which will contribute to advance the knowledge on the role of biofilms in disease and develop novel strategies to fight biofilm-associated infections.

## Data Availability

Not applicable.

## List of abbreviations

EPS: Extracellular polymeric substances
QS: Quorum sensing
